# Robust Control Based on Adaptive Neural Network for the Process of Steady Formation of Continuous Contact Force in Unmanned Aerial Manipulator

**DOI:** 10.3390/s23020989

**Published:** 2023-01-15

**Authors:** Qian Fang, Pengjun Mao, Lirui Shen, Jun Wang

**Affiliations:** School of Mechanical and Electrical Engineering, Henan University of Science and Technology, Luoyang 471000, China

**Keywords:** unmanned aerial manipulator, contact procedure, force control, robust controller, adaptive neural network

## Abstract

Contact force control for Unmanned Aerial Manipulators (UAMs) is a challenging issue today. This paper designs a new method to stabilize the UAM system during the formation of contact force with the target. Firstly, the dynamic model of the contact process between the UAM and the target is derived. Then, a non-singular global fast terminal sliding mode controller (NGFTSMC) is proposed to guarantee that the contact process is completed within a finite time. Moreover, to compensate for system uncertainties and external disturbances, the equivalent part of the controller is estimated by an adaptive radial basis function neural network (RBFNN). Finally, the Lyapunov theory is applied to validate the global stability of the closed-loop system and derive the adaptive law for the neural network weight matrix online updating. Simulation and experimental results demonstrate that the proposed method can stably form a continuous contact force and reduce the chattering with good robustness.

## 1. Introduction

In recent years, Unmanned Aerial Vehicles (UAVs) have been developing toward miniaturization and intelligence, as a kind of aerial robot. In particular, due to their Vertical Take-Off and Landing (VTOL) and hovering capabilities, multi-rotor UAVs have been used in many military or civil fields, such as photography [1], mapping [2], agriculture [3,4], inspection [5], monitoring [6,7], and data collection [8,9]. However, these applications are utilized for observation and perception, which have no direct contact with the surrounding environment. With the development of robot technology, Unmanned Aerial Manipulators (UAMs) have caught the great attention of researchers. They can supersede humans in their ability to operate on targets in hard-to-reach areas or in high-risk environments.

The research of UAM applications has gained some achievements in the last decade, including grabbing stationary or moving objects [10,11], picking up and transporting goods [12,13,14], inspecting infrastructure [15,16], and installing and retrieving equipment [17,18,19,20]. Continuous contact operations cause the UAM to lose its degree of freedom in a specific direction, so the stability control of the UAM can be more complicated. Currently, most research focuses on maintaining a stable contact force, without mentioning how this contact force is formed, and in most studies, the posture of the target is vertical and does not consider other postures; the lack of research in these areas motivates us in this study.

This study aims to design a method for contact force operation so that the force applied by the UAM can be precisely controlled from formation to stabilization. The main contributions of this paper can be summarized as follows:

1. A force/position hybrid control framework is designed for the contact process between the UAM and the inclined target. This method will generate the desired trajectory according to the formation process of the contact force.
2. A robust non-singular global fast terminal sliding mode controller (NGFTSMC) is proposed to make the system error converge rapidly during the whole process of trajectory tracking. Furthermore, since an adaptive RBFNN is used to estimate the equivalent part of the controller, the improved controller does not need any prior knowledge of the system dynamics and external disturbances, and can reduce the system chattering effectively.

The rest of this article is organized as follows. Section 2 gives an overview of the related work. Section 3 describes the dynamic model of the UAM and ameliorates the model for the contact procedure. Section 4 discusses the control strategy and designs a robust NGFTSMC, based on the adaptive RBFNN. Section 5 and Section 6 present the simulation and experimental results and analysis. Finally, the conclusion is given in Section 7.

## 2. Related Work

The tasks performed by UAMs generally fall into two categories: free-flight operation and motion-restricted operation. The free-flight operation is a typical working modality of UAM, including grasping and transporting. This operation has no contact or a brief contact with the environment, so the environment has little influence on the stability of the UAM. Therefore, the force interaction can be regarded as a significant disturbance, and the research on this operation mainly focuses on the center of gravity compensation or the grasping path planning. Haoyao Chen et al. formulated the trajectory planning for aerial grasping control as a multi-objective optimization problem, and introduced a vision-based trajectory compensation and tracking control method to address the external disturbance and the coupled affection between the manipulator and the octocopter [10]. Guangyu Zhang et al. designed a UAM platform and proposed a controller that could compensate for the shift in the system’s center of mass, caused by the movement of the manipulator [11]. To transport a payload with an unknown mass, they developed an adaptive decentralized cooperative control law that could regulate the squeeze forces and guarantee velocity convergence for all the agents [14].

Unlike free-flight operations, motion-restricted operations have a long interaction time with the targets. UAM will lose some freedom of movement if it keeps contact with and forces the target. This kind of contact task is a hybrid force and position control issue, studied by some researchers. Junhao Zeng et al. designed a new control method to decouple the position and attitude of the UAM through image features, used image-based impedance control to control the position and track contact force; they also used geometric methods to so that the developed controller could control the attitude [16]. Salua Hamaza et al. designed a small UAV for contact-based tasks, equipped with a lightweight active manipulator, capable of exerting force on the side of the aircraft to solve placement and retrieval tasks [19]. These works mainly focus on maintaining the force already applied to the target, but how to control the UAM to form the desired contact force on the target stably is an issue that cannot be ignored. In addition, the current research on the contact force of UAM is mainly aimed at the situation of the vertical surface [21,22]. However, the target’s posture in an authentic working environment is diverse, and the contact force will generate force components in three axes. Therefore, to improve the conduction efficiency and prevent the manipulator from slipping from the target, the contact force should always be perpendicular to the target.

The key to UAM tasks is precise position and attitude control. However, the UAM is a complex nonlinear system with strong coupling effects. The UAM and the target will constrain and disturb each other, which makes the UAM extremely vulnerable to system uncertainty and external disturbances. Therefore, the control of the UAM system has drawn significant attention, and a variety of control strategies have been used to improve the system’s performance. Some methods require accurate system models, including a linear quadratic regulator (LQR) [23], H∞ control [24], back-stepping control [25], model predictive control [26], etc. However, this is impossible to obtain for a real system, and the system parameters may change during the operation. As a discontinuous control technique, the sliding mode controller has the advantages of excellent robustness to nonlinear system model errors, parameter uncertainties, and external disturbance, but it has the disadvantages of infinite convergence time and theoretical chattering. The terminal sliding mode controller (TSMC) is an effective way to accomplish the finite-time convergence of system states. Yong Feng et al. improved the terminal sliding mode controller and suggested a new control strategy for nonlinear systems with fast convergence and singularity free [27]. Chattering is also a defect that cannot be omitted in the sliding mode control system. For a mechanical system, it will generate high-frequency switching signals, cause the severe wear of mechanical parts, and eventually lead to actuator damage. Samah Riache et al. proposed an adaptive non-singular terminal super-twisting sliding mode controller that accomplished finite-time convergence by avoiding any singularity problem and chattering attenuation in the existence of disturbances [28]. Jinbo Zhao et al. designed a time delay estimation–based non-singular terminal sliding mode controller to perform more precise joint position tracking. In addition, they introduced a boundary layer to reduce chattering and regulated it by fuzzy logic to achieve a swifter convergence [29].

The design of the TSMC contains detailed information regarding system parameters, but for a multi-rigid-body system UAM, the system parameters will change during the operation, and external disturbances cannot be accurately measured. Neural networks can approximate arbitrary nonlinear functions and have the ability to learn online. Therefore, neural networks are widely utilized in robotic control, combined with various controllers. Qing Guo et al. trained the unknown dynamics of the manipulator by using the radial basis function neural network (RBFNN) to substitute the pre-known dynamics in the backstepping iteration; then, they used an adaptive estimation law to optimize the estimated model online to accomplish the enhanced robustness of the neural network controllers [30]. Bin Ren et al. proposed a novel super-twisting adaptive neural network sliding mode controller. The RBFNN is used for disturbance compensation to reduce the uncertainty of the control system, and the super-twisting strategy can effectively decrease the chattering problems of the system [31]. Julian Nubert et al. suggested a novel robust setpoint tracking MPC method, which performs the dependable and secure tracking of a dynamic setpoint, while guaranteeing stability and constraint satisfaction. In addition, this MPC law is approximated by a neural network controller to diminish the MPC’s computation time drastically [32].

In this study, a simple mechanical setup, consisting of an underactuated quadrotor and one joint manipulator, is used as the experimental device, and a new control framework for the whole contact process is designed; this is performed so that the UAM can stably form the desired force with targets of any posture. In addition, the NGFTSMC is extended to UAM contact tasks for fast error convergence, and an RBF neural network estimator is used to estimate system uncertainties and external disturbances.

## 3. System Modeling

**Figure 1:**
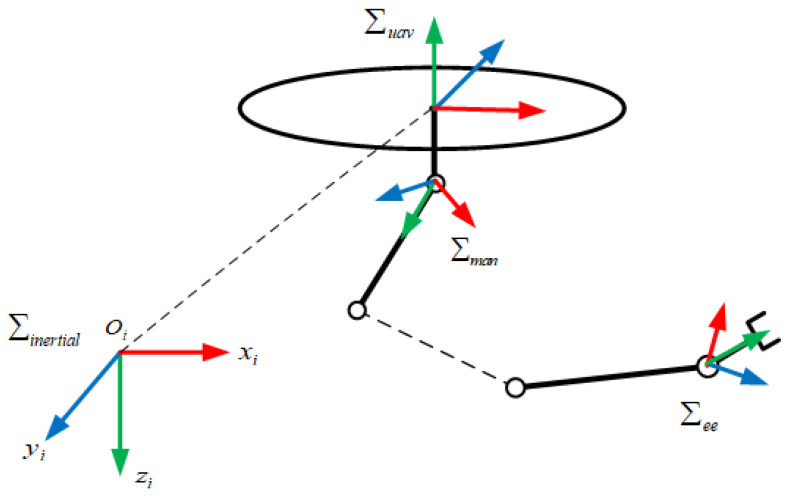
Reference frames of the UAM system.

The UAM system consists of a multi-rotor UAV and an n-link manipulator, as illustrated in Figure 1.

where $\sum_{inertial}$ is the inertial coordinate system with the origin at $O_{i}$, $\sum_{uav}$ is the UAV body coordinate system, $\sum_{man}$ is the coordinate system of the manipulator, and $\sum_{ee}$ is the coordinate system of the end effector.

The rotation matrix from the UAV body coordinate system to the inertial coordinate system can be expressed as follows:

(1) $$R_{b}^{i}=\left[\begin{array}{ccc} \cos\theta \cos\psi & \sin\phi \sin\theta \cos\psi -\cos\phi \sin\psi & \cos\phi \sin\theta \cos\psi +\sin\phi \sin\psi \\ \cos\theta \sin\psi & \sin\phi \sin\theta \sin\psi +\cos\phi \cos\psi & \cos\phi \sin\theta \sin\psi -\sin\phi \cos\psi \\ -\sin\theta & \sin\phi \cos\theta & \cos\phi \cos\theta \end{array}\right]$$

where Φ=[ϕ θ ψ] is the Euler angle of the UAV in the inertial coordinate system. The rotation matrix from the end effector of the manipulator to the UAV can be described as

(2) $$R_{e}^{b}=R_{m}^{b}R_{e}^{m}$$

where $R_{m}^{b}$ is the rotation matrix from the base of the manipulator to the UAV body, and $R_{e}^{m}$ is the rotation matrix from the end effector to the base of the manipulator. These values are determined by the structure and parameters of the UAM.

The quadrotor is an excellent platform for the manipulator due to its simple structure, low cost, and easy maintenance. The anti-torque it generates can be eliminated due to the reverse installation of the two pairs of propellers. Nevertheless, the structure of the manipulator makes it impossible to counterbalance the anti-torque generated by the joints, and more joints will generate more anti-torque. Therefore, to decrease the impact of the anti-torque and maximize the force transmission efficiency, the number of joints in the manipulator should be minimized. This study uses a quadrotor with one joint manipulator as the research object.

The contact task of the UAM is usually performed in small areas and at low speeds. Through simplification and decoupling, the dynamic equations of the quadrotor platform can be expressed as follows:

(3) $$\left\{ \begin{array}{l} \overset{\cdot \cdot }{x}=\left(cos\phi sin\theta cos\psi +sin\phi sin\psi \right)\frac{u_{1}}{m}-\frac{f_{contact-x}}{m}-d_{x}\\ \ddot{y}=\left(cos\phi sin\theta sin\psi -sin\phi cos\psi \right)\frac{u_{1}}{m}-\frac{f_{contact-y}}{m}-d_{y}\\ \ddot{z}=cos\phi cos\theta \frac{u_{1}}{m}-g-\frac{f_{contact-z}}{m}-d_{z}\\ \ddot{\phi }=\left(\frac{I_{yy}-I_{zz}}{I_{xx}}\right)\dot{\theta }\dot{\psi }-\frac{J}{I_{xx}}\dot{\theta }\Omega +\frac{ru_{2}}{I_{xx}}-d_{\phi }\\ \ddot{\theta }=\left(\frac{I_{zz}-I_{xx}}{I_{yy}}\right)\dot{\phi }\dot{\psi }-\frac{J}{I_{yy}}\dot{\phi }\Omega +\frac{ru_{3}}{I_{yy}}-d_{\theta }\\ \ddot{\psi }=\left(\frac{I_{xx}-I_{yy}}{I_{zz}}\right)\dot{\phi }\dot{\theta }+\frac{u_{4}}{I_{zz}}-d_{\psi } \end{array}\right.$$

where P=[x y z] is the position of the quadrotor, m is the mass of the aerial manipulation, g is the acceleration of gravity, r is the distance from the propeller to the center of the quadrotor, $\left[I_{xx},I_{yy},I_{zz}\right]$ is the mass moments of inertia in the x, y, and z axes, respectively, J is the rotor inertia, Ω is the total residual speed of the motor, $D=\left[d_{x}\;d_{y}\;d_{z}\;d_{\phi }\;d_{\theta }\;d_{\psi }\right]$ is the lumped disturbance including modeling errors, parameter uncertainties, and external disturbances, $\left[u_{1}\;u_{2}\;u_{3}\;u_{4}\right]$ are the input forces of the quadrotor, and $\left[f_{contact-x}\;f_{contact-y}\;f_{contact-z}\right]$ are the contact forces applied by the aerial manipulator to the target in the x, y, and z axes.

This supposes that the position and attitude of the UAM system have been adjusted and only moved in the X-Z plan. The quadrotor platform should keep hovering when there is a contact force between the UAM system and the target. According to Equation (3), ignoring the disturbance, the contact force in the hovering state can be described as:

(4) $$\{\begin{array}{l} \left(cos\phi sin\theta cos\psi +sin\phi sin\psi \right)u_{1}-f_{contact-x}=0\\ \left(cos\phi sin\theta sin\psi -sin\phi cos\psi \right)u_{1}-f_{contact-y}=0\\ cos\phi cos\theta u_{1}-mg-f_{contact-z}=0 \end{array}$$

**Figure 2:**
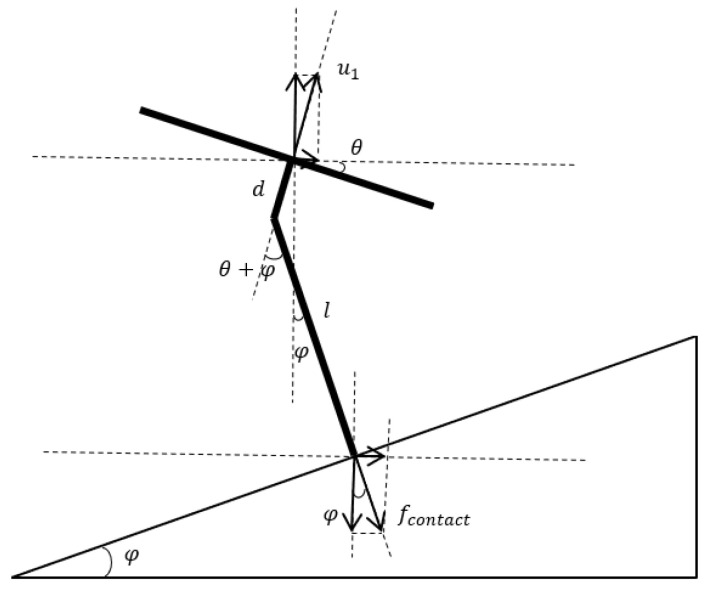
The analysis of contact force.

Assuming that the angle between the target and the horizontal is φ, and the roll and yaw of the quadrotor platform are set to zero, the analysis of the contact force is shown in Figure 2.

where l is the length of the manipulator, and d is the distance from the base of the manipulator to the center of the quadrotor. The expression of the contact force $f_{contact}$ can be derived as follows:

(5) $$\{\begin{array}{l} f_{contact}=\frac{tan\theta }{sin\varphi -cos\varphi tan\theta }mg\\ \begin{array}{l} f_{contact-x}=f_{contact}sin\varphi \\ f_{contact-y}=0\\ f_{contact-z}=f_{contact}cos\varphi \end{array} \end{array}$$

**Figure 3:**
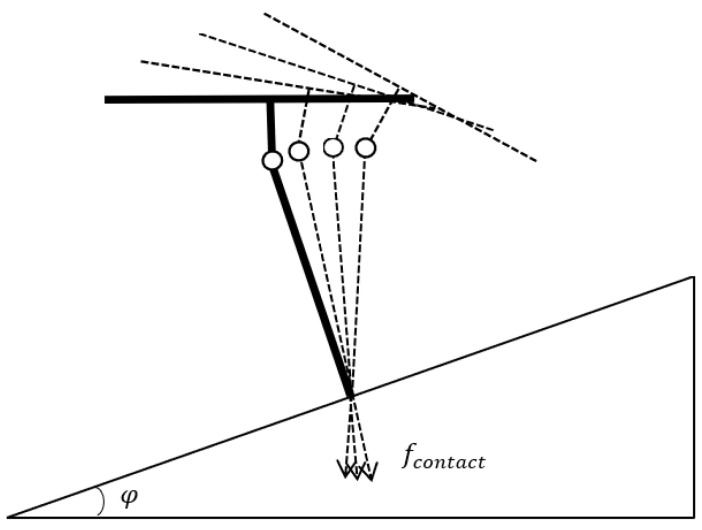
The analysis of changing the pitch angle.

It can be noticed that the contact force is decided by four parameters, m, g, φ, and θ. Since the former three parameters are constant, the contact force always corresponds to a pitch angle. However, if the pitch angle alters without adjusting the angle of the manipulator, the manipulator will no longer be perpendicular to the target and cause separation eventually. The analysis of this process is shown in Figure 3.

To ensure the manipulator is always perpendicular to the target, the angle of the rotating manipulator should be identical to the pitch angle of the quadrotor. Therefore, the desired contact force can be accomplished smoothly by adjusting the rotating and pitch angles synchronously. The desired pitch angle can be obtained from Equation (5).

(6) $$\theta_{desired}=\arctan\left(\frac{f_{desired}sin\varphi }{f_{desired}cos\varphi +mg}\right)$$

Assuming the position of the target is $P_{target}=\left[x_{t}\;y_{t}\;z_{t}\right]$, according to Figure 2, the desired position of the quadrotor platform can be obtained as

(7) $$\{\begin{array}{l} x_{d}=x_{t}-lsin\varphi +dsin\theta \\ y_{d}=y_{t}\\ z_{d}=z_{t}+lcos\varphi +dcos\theta \end{array}$$

## 4. Controller Design

By decoupling the dynamic model, the control of the UAM is equivalent to the control of the quadrotor and the manipulator, respectively. The manipulator should revolve smoothly according to the characteristics of the motor since the anti-torque cannot be eliminated. This study aims to control the pitch angle of the quadrotor platform following the rotation of the manipulator.

**Figure 4:**
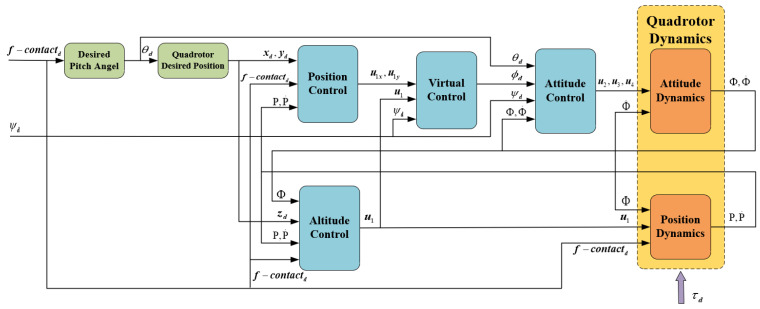
The structure of the double-loop control scheme based on contact force formation.

Since the sampling frequency of the gyroscope and accelerometer in the flight controller is distinct, this research chooses the inner and outer loop control strategy for the quadrotor platform, in which the outer loop is the position control, and the inner loop is the attitude control. With the desired force $f_{desired}$, the desired pitch angle $\theta_{d}$ and position $\left[x_{d}\;y_{d}\;z_{d}\right]$ can be calculated according to Equations (6) and (7). However, the quadrotor is an under-actuated system, and its horizontal position [x y] and Euler angle [ϕ θ] are coupled, so it is necessary to design the virtual controller of [x y] to derive the expected value $\phi_{d}$. Then, with the desired pitch angle $\theta_{d}$, the roll and pitch controller can be designed. As the UAM’s altitude z and yaw angle ψ are not coupled to other states, their controllers can be designed directly. The structure of the control system is depicted in Figure 4.

### 4.1. Altitude Control

Define the altitude error as

(8) $$e_{z}=z-z_{d}$$

where $z_{d}$ is the desired altitude. The non-singular fast terminal sliding surface function is designed as

(9) $$s={\dot{e}}_{z}+\alpha_{1}e_{z}+\beta_{1}{\left|e_{z}\right|}^{\mu_{1}/v_{1}}sgn\left(e_{z}\right)$$

where $\alpha_{1}>0$, $\beta_{1}>0$, $\mu_{1}$ and $\nu_{1}$ are positive odd integers that satisfy $\mu_{1}<\nu_{1}$. $sgn\left(e_{z}\right)$ is the switching function described as follows:

(10) $$sgn\left(e_{z}\right)=\{\begin{array}{c} 1,e_{z}>0\\ 0,e_{z}=0\\ -1,e_{z}<0 \end{array}$$

Taking the time derivative of Equation (9), $\dot{s}$ can be deduced as

(11) $$\dot{s}={\ddot{e}}_{z}+\alpha_{1}{\dot{e}}_{z}+\frac{\beta_{1}\mu_{1}}{v_{1}}e_{z}^{\left(\mu_{1}-v_{1}\right)/v_{1}}{\dot{e}}_{z}sgn\left(e_{z}\right)$$

Equation (11) can be rewritten as follows by substituting Equation (3):

(12) $$\dot{s}=\frac{cos\phi cos\theta }{m}u_{1}-g-\frac{f_{contact-z}}{m}-d_{z}-{\ddot{z}}_{d}+\alpha_{1}{\dot{e}}_{z}+\frac{\beta_{1}\mu_{1}}{v_{1}}e_{z}^{\left(\mu_{1}-v_{1}\right)/v_{1}}{\dot{e}}_{z}sgn\left(e_{z}\right)$$

According to Equation (12), the equivalent control input is designed as

(13) $$u_{eq}=\frac{m}{cos\phi cos\theta }\left({\ddot{z}}_{d}+g+\frac{f_{contact-z}}{m}+d_{z}-\alpha_{1}{\dot{e}}_{z}-\frac{\beta_{1}\mu_{1}}{v_{1}}e_{z}^{\left(\mu_{1}-v_{1}\right)/v_{1}}{\dot{e}}_{z}sgn\left(e_{z}\right)\right)$$

In order to achieve the swift arrival of the system state to the sliding surface in finite time, the switching control input is defined as

(14) $$u_{sw}=-\frac{m}{cos\phi cos\theta }\left(\alpha_{2}s+\beta_{2}s^{\mu_{2}/\nu_{2}}\right)$$

where $\alpha_{2}>0$, $\beta_{2}>0$, $\mu_{2}$ and $\nu_{2}$ are positive odd integers that satisfy $\mu_{2}<\nu_{2}$. Then the NGFTSMC can be expressed as follows:

(15) $$u_{1}=u_{eq}+u_{sw}=\frac{m}{cos\phi cos\theta }\left({\ddot{z}}_{d}+g+\frac{f_{contact-z}}{m}+d_{z}-\alpha_{1}{\dot{e}}_{z}-\frac{\beta_{1}\mu_{1}}{v_{1}}e_{z}^{\left(\mu_{1}-v_{1}\right)/v_{1}}{\dot{e}}_{z}sgn\left(e_{z}\right)-\alpha_{2}s-\beta_{2}s^{\frac{\mu_{2}}{\nu_{2}}}\right)$$

Define the Lyapunov function as follows:

(16) $$V_{1}=\frac{1}{2}s^{2}\geq 0$$

Taking the time derivative of Equation (16), ${\dot{V}}_{1}$ can be deduced as

(17) $${\dot{V}}_{1}=s\dot{s}=-\left(\alpha_{2}s^{2}+\beta_{2}s^{\left(\mu_{2}+v_{2}\right)/v_{2}}\right)$$

where $\mu_{2}+v_{2}$ is the even number, so ${\dot{V}}_{1}\leq 0$ and the system is stable.

The designed equivalent controller contains precise model information and external disturbances. Nevertheless, these values cannot be obtained accurately for an actual system, and the system parameters may alter during operation. To improve the system’s performance, we introduce the RBFNN to estimate the equivalent controller. The estimated equivalent controller is more similar to the actual system, especially when the model parameters change.

Define the input of the RBFNN as $X={\left(\begin{array}{cc} e_{z} & {\dot{e}}_{z} \end{array}\right)}^{T}$, and the approximate equivalent controller output is

(18) $${\hat{u}}_{eq}={\hat{W}}^{T}h\left(X\right)$$

where $\hat{W}$ is the weight and h(X) is the nonlinear mapping of the RBFNN. The equivalent control in Equation (13) is an ideal value, which can be described as

(19) $$u_{eq}^{*}=W^{T}h\left(X\right)+\varepsilon$$

where W is the ideal weight, and ε is a small positive real number that represents the approximation error of the neural network and non-linear uncertainty function. Define the error generated by the neural network estimation as

(20) $$\begin{array}{ll} {\tilde{u}}_{eq} & =u_{eq}-{\hat{u}}_{eq}\\ & =W^{T}h\left(X\right)+\varepsilon -{\hat{W}}^{T}h\left(X\right)\\ & ={\tilde{W}}^{T}h\left(X\right)+\varepsilon \end{array}$$

where ${\tilde{W}}^{T}=W^{T}-{\hat{W}}^{T}$, and Equation (16) can be rewritten as

(21) $$\begin{array}{ll} \dot{s}=\frac{cos\phi cos\theta }{m} & \left({\hat{u}}_{eq}+u_{sw}\right)-\frac{cos\phi cos\theta }{m}u_{eq}\\ & =-\alpha_{2}s-\beta_{2}s^{\frac{\mu_{2}}{\nu_{2}}}+\frac{cos\phi cos\theta }{m}\left({\hat{u}}_{eq}-u_{eq}\right)\\ & =-\alpha_{2}s-\beta_{2}s^{\frac{\mu_{2}}{\nu_{2}}}-\frac{cos\phi cos\theta }{m}\left({\tilde{W}}^{T}h\left(X\right)+\varepsilon \right) \end{array}$$

The Lyapunov function is selected as

(22) $$V_{2}=\frac{1}{2}s^{2}+\frac{1}{2\xi }{\tilde{W}}^{T}\tilde{W}\geq 0$$

Equation (22) can be rewritten as follows by taking the time derivative:

(23) $$\begin{array}{ll} {\dot{V}}_{2}=s\dot{s}+\frac{1}{\xi }{\tilde{W}}^{T}\dot{\tilde{W}} & =-\alpha_{2}s^{2}-\beta_{2}s^{\frac{\mu_{2}+v_{2}}{\nu_{2}}}-\frac{cos\phi cos\theta }{m}\varepsilon s-\frac{cos\phi cos\theta }{m}s{\tilde{W}}^{T}h\left(X\right)+\frac{1}{\xi }{\tilde{W}}^{T}\dot{\tilde{W}}\\ & =-\alpha_{2}s^{2}-\left(\beta_{2}+\frac{cos\phi cos\theta }{ms^{\mu_{2}/v_{2}}}\varepsilon \right)s^{\frac{\mu_{2}+v_{2}}{\nu_{2}}}+{\tilde{W}}^{T}\left(\frac{1}{\xi }\dot{W}-\frac{cos\phi cos\theta }{m}sh\left(X\right)\right) \end{array}$$

**Figure 5:**
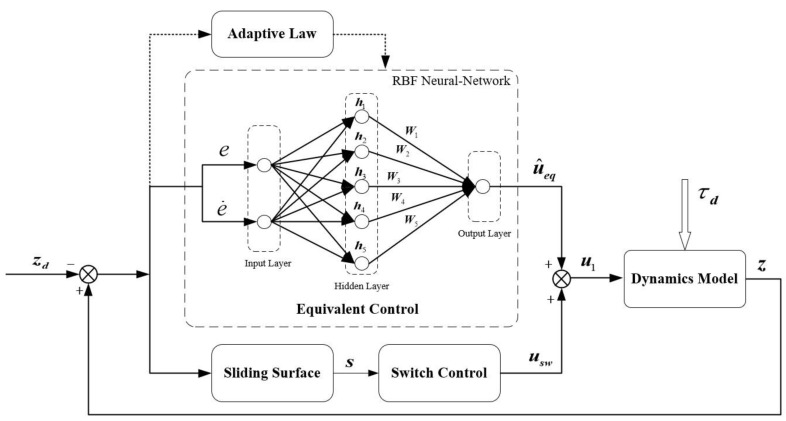
The structure of the NGFTSMC for altitude control, based on the RBFNN observer.

Define the design adaptation law as

(24) $$\dot{W}=\frac{cos\phi cos\theta }{m}\xi sh\left(X\right)$$

under the circumstance that $\beta_{2}>-\frac{cos\phi cos\theta }{ms^{\mu_{2}/v_{2}}}\varepsilon$, ${\dot{V}}_{2}\leq 0$, and the system is stable. The structure of the NGFTSMC, based on the RBFNN, is shown in Figure 5.

### 4.2. Horizontal Position Control

The horizontal subsystem [x y] is an underactuated system, so set the virtual input force as follows according to Equation (3):

(25) $$\{\begin{array}{c} u_{x}=\left(cos\phi sin\theta cos\psi +sin\phi sin\psi \right)u_{1}\\ u_{y}=\left(cos\phi sin\theta sin\psi -sin\phi cos\psi \right)u_{1} \end{array}$$

By using the above controller design method, the controller of virtual input force can be devised as

(26) $$u_{x}=m\left({\ddot{x}}_{d}+\frac{sin\varphi }{m}f_{contact}+d_{x}-\alpha_{1}{\dot{e}}_{x}-\frac{\beta_{1}\mu_{1}}{v_{1}}e_{x}^{\left(\mu_{1}-v_{1}\right)/v_{1}}{\dot{e}}_{x}sgn\left(e_{x}\right)-\alpha_{2}s_{x}-\beta_{2}s_{x}^{u_{2}/v_{2}}\right)$$

(27) $$u_{y}=m\left({\ddot{y}}_{d}+d_{y}-\alpha_{1}{\dot{e}}_{y}-\frac{\beta_{1}\mu_{1}}{v_{1}}e_{y}^{\left(\mu_{1}-v_{1}\right)/v_{1}}{\dot{e}}_{y}sgn\left(e_{y}\right)-\alpha_{2}s_{y}-\beta_{2}s_{y}^{u_{2}/v_{2}}\right)$$

After being estimated by the RBFNN, the control inputs and the corresponding adaptation laws are listed as

(28) $$\{\begin{array}{l} {\hat{u}}_{x}={\hat{W}}_{x}^{T}h_{x}-m\left(\alpha_{2}s_{x}+\beta_{2}s_{x}^{u_{2}/v_{2}}\right)\\ {\dot{\hat{W}}}_{x_{i}}=\frac{\xi s_{x}h_{x_{i}}}{m} \end{array}$$

(29) $$\{\begin{array}{l} {\hat{u}}_{y}={\hat{W}}_{y}^{T}h_{y}-m\left(\alpha_{2}s_{y}+\beta_{2}s_{y}^{u_{2}/v_{2}}\right)\\ {\dot{\hat{W}}}_{y_{i}}=\frac{\xi s_{y}h_{y_{i}}}{m} \end{array}$$

### 4.3. Attitude Control

As an uncoupled variable, the desired yaw value $\psi_{d}$ is supposed to be given. The desired pitch value $\theta_{d}$ is determined by the contact force $f_{contact}$. Therefore, the desired roll value $\phi_{d}$ can be calculated by solving Equation (25).

(30) $$\phi_{d}=\arcsin\left(\frac{sin\psi_{d}u_{x}-cos\psi_{d}u_{y}}{U}\right)$$

The attitude input is proposed by the previously designed control strategy as

(31) $$u_{2}=\frac{I_{xx}}{r}\left({\ddot{\phi }}_{d}-\frac{I_{yy}-I_{zz}}{I_{xx}}\dot{\theta }\dot{\psi }+\frac{J}{I_{xx}}\dot{\theta }\Omega +\frac{\tau_{contact-\phi }}{I_{xx}}+d_{\phi }-\alpha_{1}{\dot{e}}_{\phi }-\frac{\beta_{1}\mu_{1}}{v_{1}}e_{\phi }^{\left(\mu_{1}-v_{1}\right)/v_{1}}{\dot{e}}_{\phi }sgn\left(e_{\phi }\right)-\alpha_{2}s-\beta_{2}s^{\frac{\mu_{2}}{\nu_{2}}}\right)$$

(32) $$u_{3}=\frac{I_{yy}}{r}\left({\ddot{\theta }}_{d}-\frac{I_{zz}-I_{xx}}{I_{yy}}\dot{\psi }\dot{\phi }+\frac{J}{I_{yy}}\dot{\phi }\Omega +\frac{\tau_{contact-\theta }}{I_{xx}}+d_{\theta }-\alpha_{1}{\dot{e}}_{\theta }-\frac{\beta_{1}\mu_{1}}{v_{1}}e_{\theta }^{\left(\mu_{1}-v_{1}\right)/v_{1}}{\dot{e}}_{\theta }sgn\left(e_{\theta }\right)-\alpha_{2}s-\beta_{2}s^{\frac{\mu_{2}}{\nu_{2}}}\right)$$

(33) $$u_{4}=\frac{I_{zz}}{r}\left({\ddot{\psi }}_{d}-\frac{I_{xx}-I_{yy}}{I_{zz}}\dot{\phi }\dot{\theta }+\frac{\tau_{contact-\psi }}{I_{xx}}+d_{\psi }-\alpha_{1}{\dot{e}}_{\psi }-\frac{\beta_{1}\mu_{1}}{v_{1}}e_{\psi }^{\left(\mu_{1}-v_{1}\right)/v_{1}}{\dot{e}}_{\psi }sgn\left(e_{\psi }\right)-\alpha_{2}s-\beta_{2}s^{\frac{\mu_{2}}{\nu_{2}}}\right)$$

After being estimated by the RBFNN, the control inputs and the corresponding adaptation laws are detailed as

(34) $$\{\begin{array}{l} {\hat{u}}_{\phi }={\hat{W}}_{\phi }^{T}h_{\phi }-\frac{I_{xx}}{r}\left(\alpha_{2}s_{\phi }+\beta_{2}s_{\phi }^{u_{2}/v_{2}}\right)\\ {\dot{\hat{W}}}_{\phi_{i}}=\frac{r}{I_{xx}}\xi s_{\phi }h_{\phi_{i}} \end{array}$$

(35) $$\{\begin{array}{l} {\hat{u}}_{\theta }={\hat{W}}_{\theta }^{T}h_{\theta }-\frac{I_{yy}}{r}\left(\alpha_{2}s_{\theta }+\beta_{2}s_{\theta }^{u_{2}/v_{2}}\right)\\ {\dot{\hat{W}}}_{\theta_{i}}=\frac{r}{I_{yy}}\xi s_{\theta }h_{\theta_{i}} \end{array}$$

(36) $$\{\begin{array}{l} {\hat{u}}_{\psi }={\hat{W}}_{\psi }^{T}h_{\psi }-\frac{I_{zz}}{r}\left(\alpha_{2}s_{\psi }+\beta_{2}s_{\psi }^{u_{2}/v_{2}}\right)\\ {\dot{\hat{W}}}_{\psi_{i}}=\frac{r}{I_{zz}}\xi s_{\psi }h_{\psi_{i}} \end{array}$$

## 5. Simulation

In this section, numerical simulations are performed to verify whether the proposed controller can be successfully extended to the contact force control of the UAM. Since many researchers have already confirmed the advantages of the NGFTSMC [33,34], this paper compares the proposed controller with the NGFTSMC in simulations. 

### 5.1. Simulation Settings

The UAM system utilized in the simulation is a quadrotor equipped with one joint manipulator, as depicted in Figure 4. The system parameters are displayed in Table 1.

In the simulation, the target position is supposed to be known. The initial state of the UAM system is that the quadrotor platform hovers stably, and the end effector of the manipulator is in vertical contact with the target without generating contact force. First, the contact force is gradually increased to 10 N within 2 s, and then the contact force is maintained for 2 s to 4 s. The parameters of the proposed NGFTSMC, based on the RBFNN observer, are displayed in Table 2. The selection of all the parameters is obtained through multiple simulations that can improve the system’s performance. To test the system’s robustness, assume the lumped disturbance added to the system model, which on the position model is $\tau_{d\_P}=10rand\left(1\right)+10\cos\left(0.1t\right)$ and on the attitude model is $\tau_{d\_\Phi }=5rand\left(1\right)+5\sin\left(0.1t\right)$.

### 5.2. Simulation Results and Analysis

**Figure 6:**
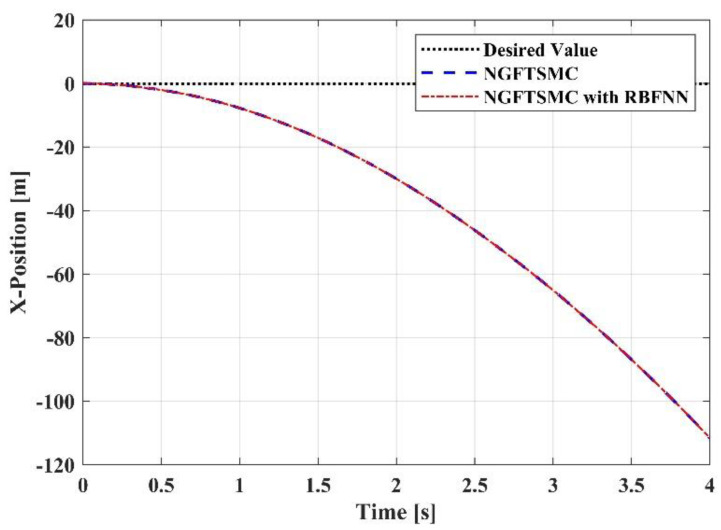
The trajectory tracking results in X-Position.

**Figure 7:**
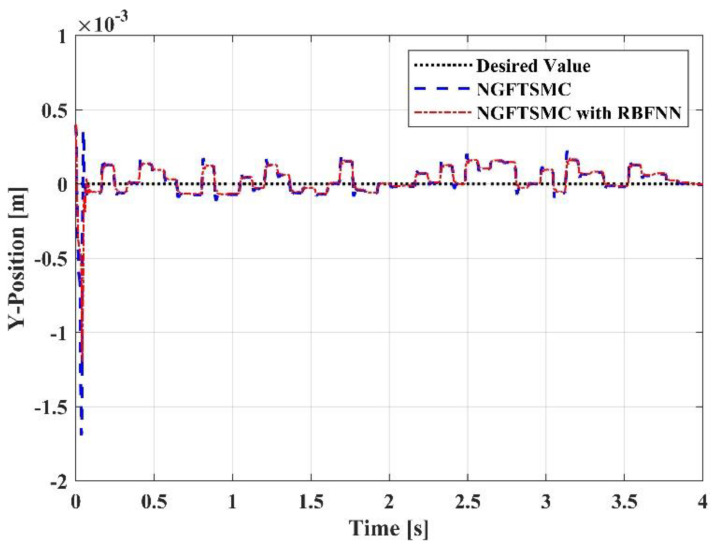
The trajectory tracking results in Y-Position.

**Figure 8:**
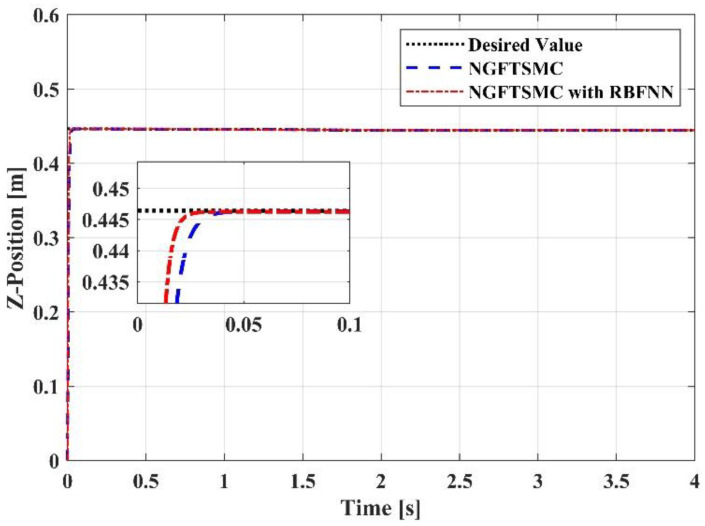
The trajectory tracking results in Z-Position.

Figure 6 depicts the trajectory tracking of the controllers in the *x*-axis direction. After contact, the reaction force generated by the target will theoretically offset the force exerted by the end effector, so that the aerial manipulation system is limited in the *x*-axis direction. However, in the simulation, the target will not generate a reaction force, and the system has no limit in the *x*-axis direction. Therefore, it can be observed that the trajectories of the systems controlled by the two methods in the *x*-axis direction are divergent. The trajectory of the system controlled by the proposed controller can track the trajectory of the system controlled by the NGFTSMC accurately, and the error of the two is within 0.01 m. Figure 7 illustrates the trajectory tracking of the controllers in the *y*-axis direction. Due to the random disturbances added to the system, some fluctuations appear in the two systems. The trajectory of the RBFNN-based NGFTSMC system is similar to the NGFTSMC system but smoother, which indicates that the proposed controller has a superior anti-disturbance performance. Figure 8 describes the trajectory tracking of the controllers in the *z*-axis direction. It can be seen that the systems controlled by both controllers can track the desired trajectory of the system. Nonetheless, the system controlled by the NGFTSMC needs 0.04 s to reach the desired trajectory, while the RBFNN-based NGFTSMC only needs 0.03 s, indicating that the proposed controller has a better performance in convergence speed.

**Figure 9:**
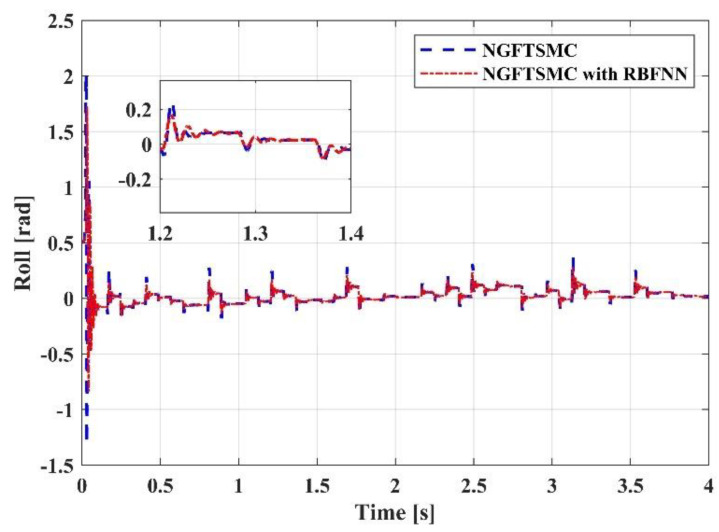
The trajectory tracking results in roll angel.

**Figure 10:**
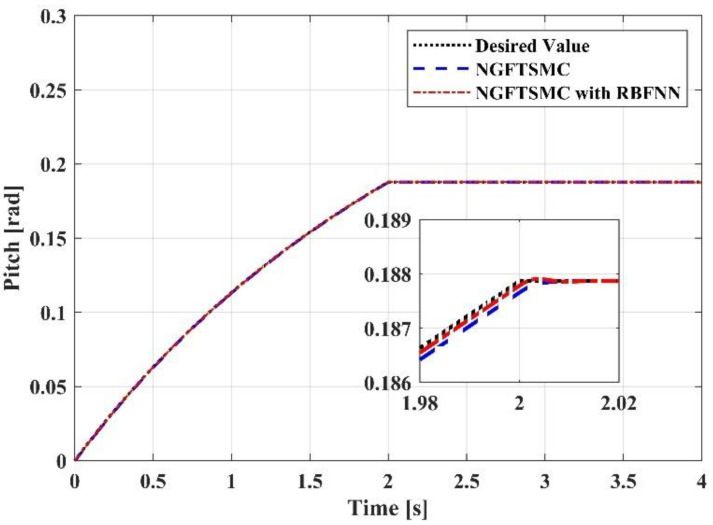
The trajectory tracking results in pitch angel.

**Figure 11:**
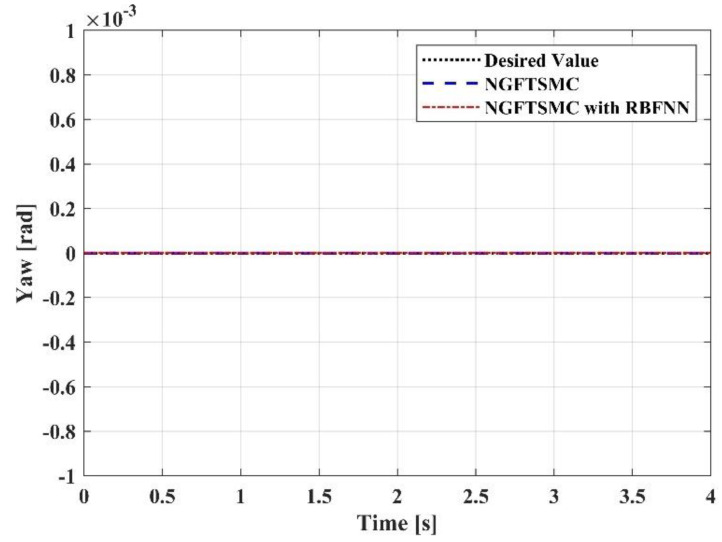
The trajectory tracking results in yaw angel.

Figure 9, Figure 10 and Figure 11 portray the variation in the system Euler angles over time. Since the roll angle is coupled with the motion in the *y*-axis direction, the changing pattern in the roll angle should be similar to the trajectory of the system in the *y*-axis direction. It can be noticed from Figure 9 that the variation in the roll angle of the RBFNN-based NGFTSMC system is slighter than that of the NGFTSMC system. The compensation of the neural network can decrease the influence of random disturbances, and the simulation results are compatible with the analysis. The pitch angle is coupled with the motion of the system in the *x*-axis direction. Since the trajectory on the *x*-axis is divergent, the acceleration always exists, and the desired value of the pitch angle is not zero. Therefore, as shown in Figure 10, the proposed controller can better track the system’s desired trajectory and converge to the equilibrium state faster than the NGFTSMC. Since the yaw angle is not coupled with other states, the system can control this state directly, which is advantageous to the system. This characteristic allows both systems controlled by the NGFTSMC and the RBFNN-based NGFTSMC in Figure 11 to exhibit outstanding control performance and effectively diminish the chattering of the system.

**Figure 12:**
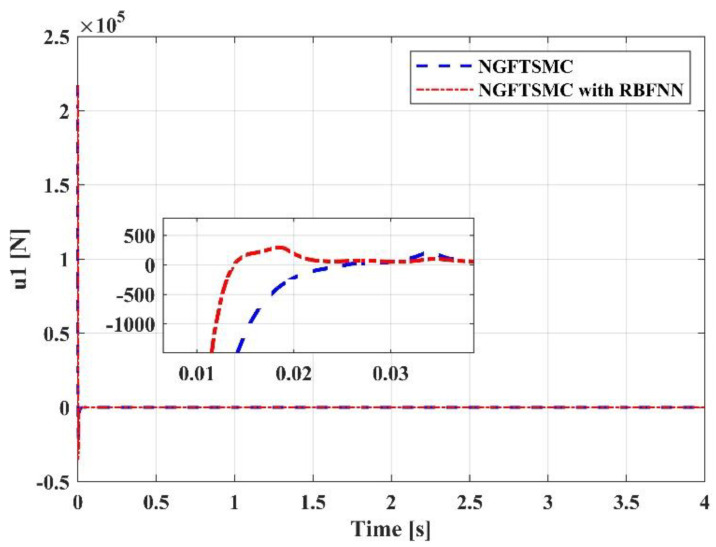
The performance of the control input $u_{1}$.

**Figure 13:**
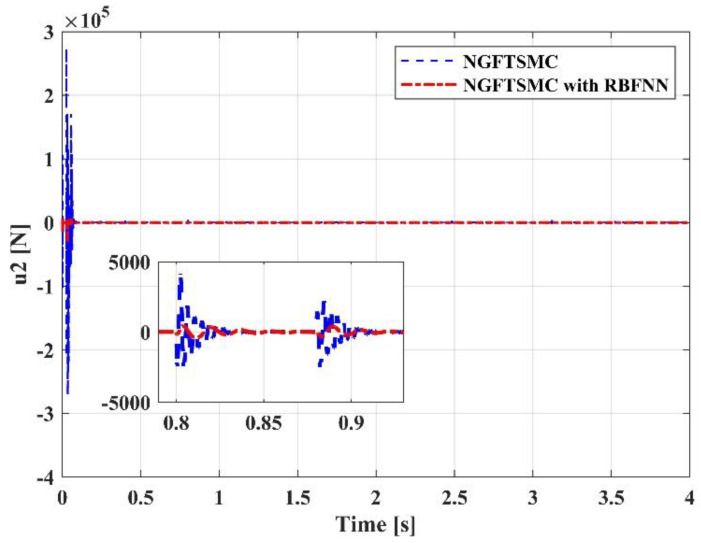
The performance of the control input $u_{2}$.

**Figure 14:**
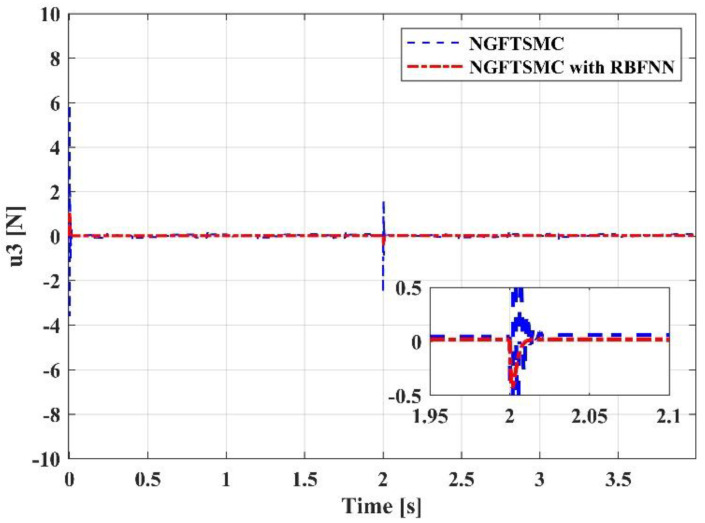
The performance of the control input $u_{3}$.

**Figure 15:**
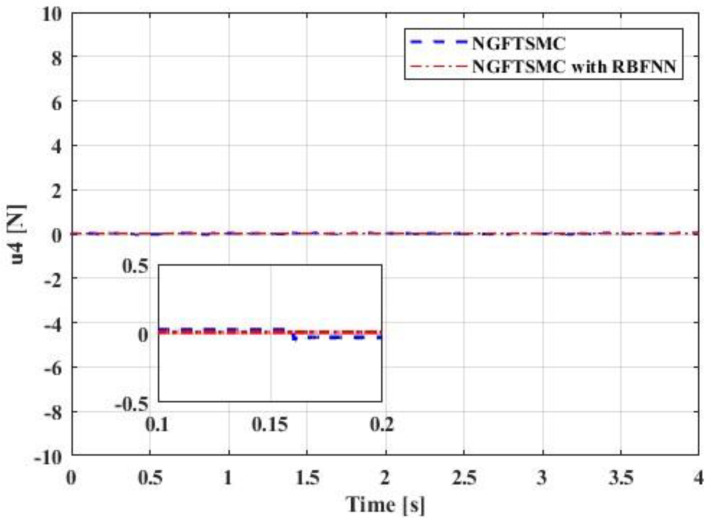
The performance of the control input $u_{4}$.

Figure 12 displays the performance of the control input $u_{1}$. The response speed of the system controlled by the proposed controller is swifter than that of the system controlled by the NGFTSMC. Since $u_{1}$ represents the thrust of the system, it mainly affects the motion on the *z*-axis, so its performance is consistent with the trajectory tracking results in Figure 8. Figure 13, Figure 14 and Figure 15 are the traces of the system torque output by the controllers over time. Distinct from Figure 12, the torque output of the system, controlled by the NGFTSMC and based on the RBFNN, is around one-sixth of that of the NGFTSMC-controlled system; in addition, the frequency and amplitude of the fluctuations are less. This performance will reduce the wear of the mechanical components and have an eminent significance for the proposed controller application in the actual system.

**Figure 16:**
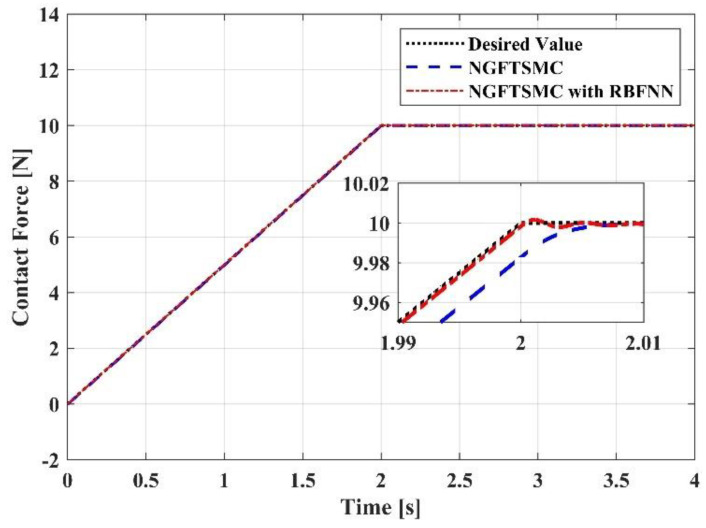
Contact force generated by the aerial manipulation.

This research aims to make the UAM system generate a constant and stable contact force on the target. The model analysis indicates that the magnitude of the contact force map to the dimension of the pitch angle of the UAV platform, and the contact force generated by the UAM system on the target can be calculated according to Equation (5). As shown in Figure 16, the trajectory of the contact force has two phases. In the increasing phase, the tracking error of the RBFNN-based NGFTSMC system is approximately 0.002 N, and the tracking error of the NGFTSMC system is approximately 0.017 N. It can be seen that the performance of the proposed controller is significantly better than that of the NGFTSMC system. Then, in the contact force maintenance phase, the system controlled by the proposed controller converges faster than that controlled by the NGFTSMC. The designed controller is insensitive to the lumped disturbance because of the compensation of the RBFNN. In addition, it does not require any information about the dynamic model, so it exhibits remarkable robustness; this is because, no matter how the system parameters alter the performance of the system will not be altered.

## 6. Experiment

**Figure 17:**
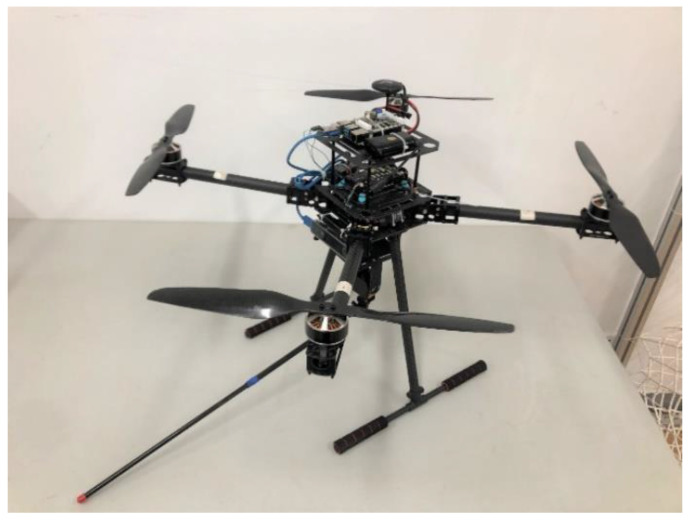
The UAM system.

**Figure 18:**
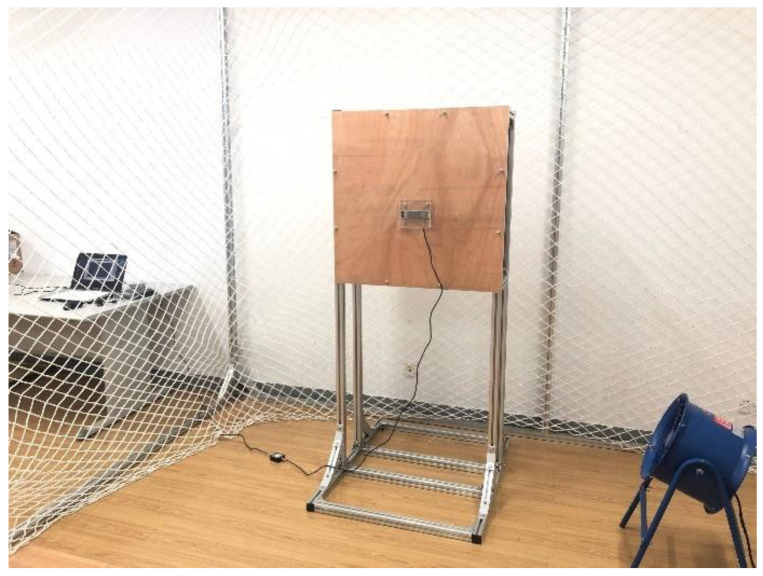
The pressure information acquisition system.

In this section, in order to substantiate the effectiveness of the proposed control scheme, an UAM system and a pressure information acquisition system are built, and an experimental flight test of the contact force formation is performed. As shown in Figure 17, the UAM system consists of a 680 mm quadrotor platform, a single-joint manipulator, and a Raspberry Pi 4 b as the onboard computer. The pressure information acquisition system in Figure 18 comprises a pressure sensor, a signal amplifier, a data acquisition card, and a host computer. The measured pressure signals are transmitted to the data acquisition card through the signal amplifier, the data acquisition card sends the collected data to the host computer, and then the host computer saves and displays the data through the software. To test the robustness of the designed controller, we place a fan beside the pressure information acquisition system, which is regarded as the external disturbance to the UAM system when performing the contact operation.

**Figure 19:**
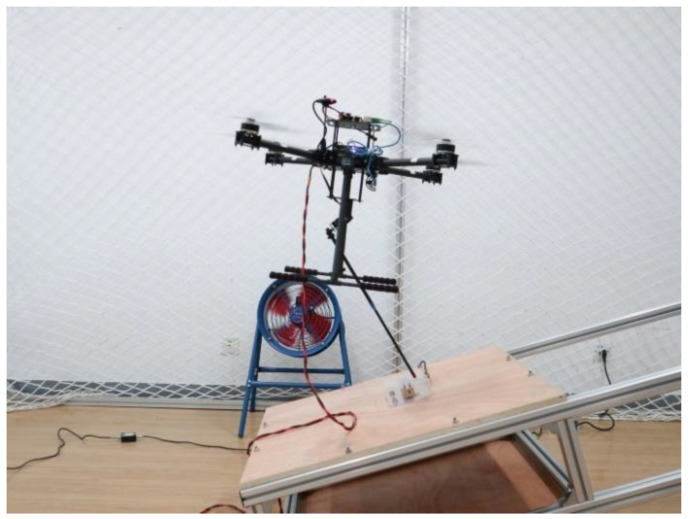
The contact force formation experiment of the UAM system.

In the experiments, the initial state of the quadrotor platform is adjusted to hover at the operating position, and the manipulator is perpendicular to the target as close as possible. Then, the autopilot is activated to perform the contact operation. The UAM system gradually increases the contact force to 10 N in 0 to 5 s and endeavors to maintain the 10 N contact force stably in 5 to 10 s. Figure 19 displays the experimental process in which the UAM slowly generates contact force with the target on the slope.

**Figure 20:**
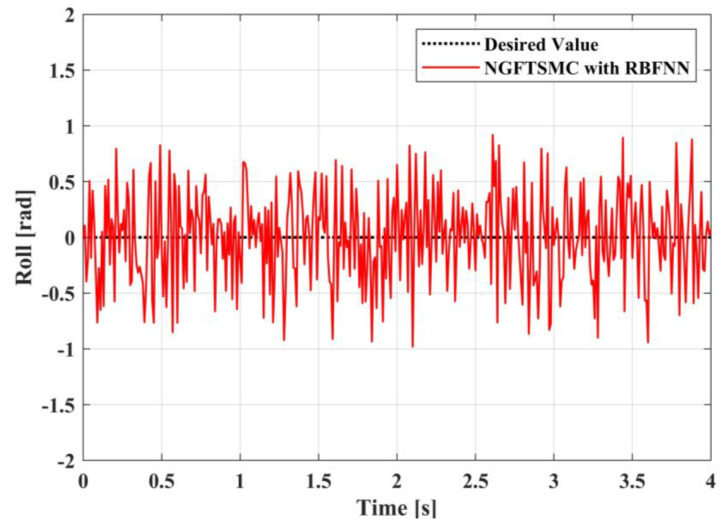
The experimental results in roll angel.

**Figure 21:**
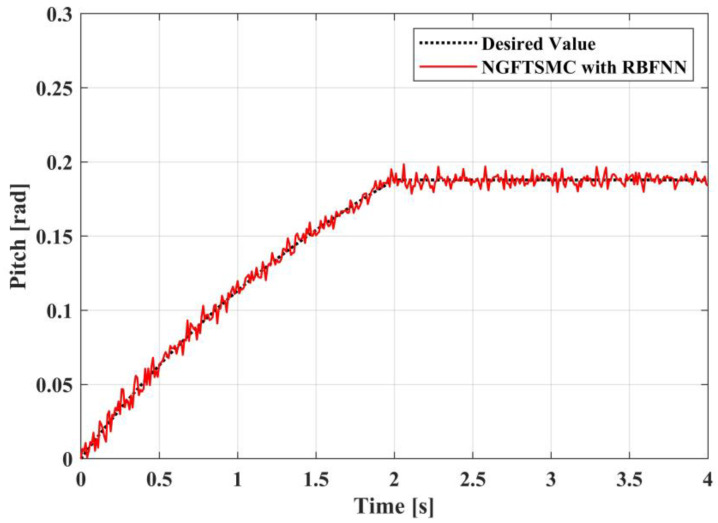
The experimental results in pitch angel.

**Figure 22:**
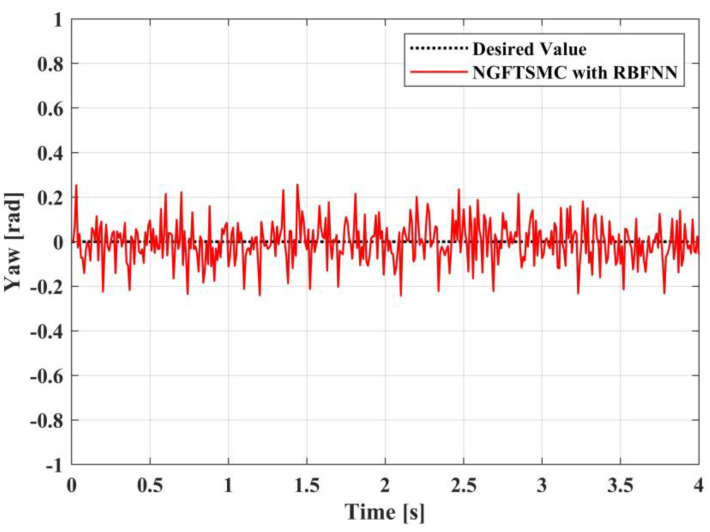
The experimental results in yaw angel.

**Figure 23:**
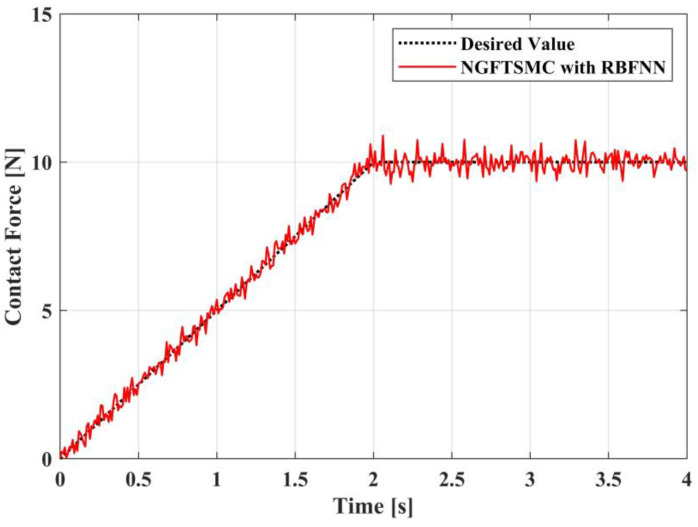
The contact force results.

**Figure 24:**
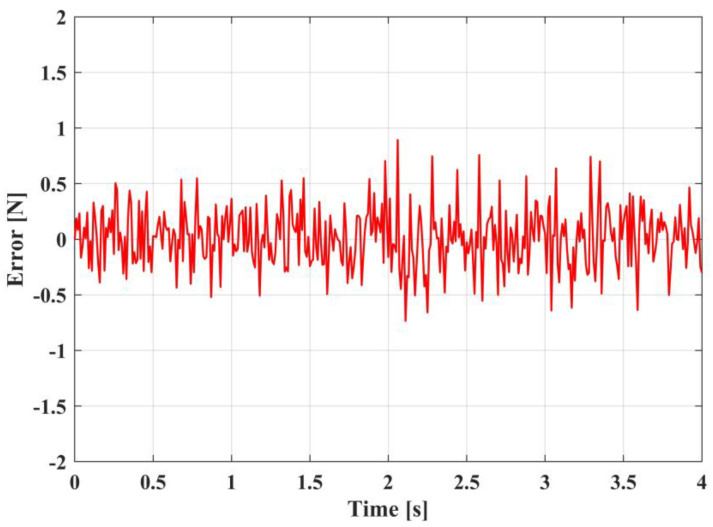
The contact force errors.

Figure 20, Figure 21 and Figure 22 exhibit the variation in the system Euler angles over time in the experiment. It can be seen that, because the four motors cannot be completely synchronized, the Euler angle changes in a small amplitude. However, after the adjustment of the system controller, the system is basically stable without significant fluctuations. Therefore, the experimental results in Figure 23 indicate that the proposed controller can effectively drive the UAM to gradually increase the contact force to the desired value and retain this contact force effectively. Moreover, Figure 24 shows that the error in the contact force formation process is smaller than in the contact force maintenance process, but the control system can keep the error of contact force within 0.6 N during the experiment.

## 7. Conclusions

In this paper, we analyzed the characteristics of the contact process between the UAM and the target, and designed a new force/position hybrid control framework to stabilize the UAM system during the formation of the contact force. Based on this framework, we obtained the desired system state and proposed an NGFTSMC with an RBFNN estimator to track the position and attitude trajectory; this was calculated by the system dynamics. According to the Lyapunov theory, the stability requirement of the system was obtained and the adaptive law for the neural network weight matrix was derived. Numerical simulations were carried out to prove the ascendency of the proposed controller compared with the NGFTSMC. The results revealed that the proposed controller tracks the desired trajectory faster than the NGFTSMC and yields the system output more smoothly by reducing the system chattering. In addition, by estimating the equivalent part in the RBFNN, the NGFTSMC no longer requires the dynamic model of the system, which can eliminate the impediments brought by system uncertainties. Finally, the experiments verify the performance and effectiveness of the proposed controller.

In this work, the target position is supposed to be known, and we drive the quadrotor to leave it hovering over the target. Future work will focus on target detection and increase the adaptability of the system to different working environments by combining it with the kinematics of the flying manipulator. In addition, differentiated end effectors also need to be considered.

## Figures and Tables

**Table 1:** Parameters of the aerial manipulator simulation.

Parameter	Value	Units
m	1.8	kg
g	9.8	$m\cdot s^{-2}$
$I_{xx}$	1.24	$\mathrm{kg}\cdot m^{2}$
$I_{yy}$	1.24	$\mathrm{kg}\cdot m^{2}$
$I_{zz}$	2.48	$\mathrm{kg}\cdot m^{2}$
d	0.1	m
l	0.4	m
$P_{target}$	(0, 0, 0)	m
φ	30	°
$f_{contact}$	10	N

**Table 2:** Parameters of NGFTSMC based on RBFNN observer.

Parameter in the Position Loop	Value	Parameter in the Attitude Loop	Value	Parameter of the RBFNN Observer	Value
$\alpha_{1}$	200	$\alpha_{1}$	1000	c	[−1.0−0.5 0 0.5 1]
$\beta_{1}$	10	$\beta_{1}$	60	b	10
$\mu_{1}$	3	$\mu_{1}$	3	W	[0 0 0 0 0]
$\nu_{1}$	5	$\nu_{1}$	5	ξ	0.1
$\alpha_{2}$	800	$\alpha_{2}$	400		
$\beta_{2}$	100	$\beta_{2}$	100		
$\mu_{2}$	3	$\mu_{2}$	3		
$\nu_{2}$	5	$\nu_{2}$	5		

## Data Availability

The data used to support the findings of this study are included within the article.

## Nomenclature

UAV	Unmanned Aerial Vehicle
UAM	Unmanned Aerial Manipulator
TSMC	Terminal Sliding Mode Controller
RBFNN	Radial Basis Function Neural Network
NGFTSMC	Non-singular Global Fast Terminal Sliding Mode Controller
$\sum_{inertial}$	The inertial coordinate system
$\sum_{uav}$	The UAV body coordinate system
$\sum_{man}$	The coordinate system of the manipulator
$\sum_{ee}$	The coordinate system of the end effector
$R_{b}^{i}$	The rotation matrix from the UAV body coordinate system to the inertial coordinate system
Φ=[ϕ θ ψ]	The Euler angle of the UAV in the inertial coordinate system
$R_{e}^{b}$	The rotation matrix from the end effector of the manipulator to the UAV
$R_{m}^{b}$	The rotation matrix from the base of the manipulator to the UAV body
$R_{e}^{m}$	The rotation matrix from the end effector to the base of the manipulator
P=[x y z]	The position of the quadrotor
m	The mass of the aerial manipulator
g	The acceleration of gravity
$\left[I_{xx},I_{yy},I_{zz}\right]$	The mass moments of inertia in the x, y, and z axes
J	The rotor inertia
Ω	The total residual speed of the motor
$D=\left[d_{x}\;d_{y}\;d_{z}\;d_{\phi }\;d_{\theta }\;d_{\psi }\right]$	The lumped disturbance including modeling errors, parameter uncertainties, and external disturbances
$\left[u_{1}\;u_{2}\;u_{3}\;u_{4}\right]$	The input forces of the quadrotor
$\left[f_{contact-x}\;f_{contact-y}\;f_{contact-z}\right]$	The contact forces applied by the aerial manipulator to the target in the x, y, and z axes
φ	The angle between the target and the horizontal
l	The length of the manipulator
d	The distance from the base of the manipulator to the center of the quadrotor
$f_{contact}$	The contact force
$P_{target}=\left[x_{t}\;y_{t}\;z_{t}\right]$	The position of the target
$\left[x_{d}\;y_{d}\;z_{d}\right]$	The desired position
$\left[\phi_{d}\;\theta_{d}\;\psi_{d}\right]$	The desired attitude
$\left[e_{x}\;e_{y}\;e_{z}\;e_{\phi }\;e_{\theta }\;e_{\psi }\right]$	The tracking errors
s	The sliding surface function
$\alpha_{1},\beta_{1},\mu_{1},\nu_{1},\alpha_{2},\beta_{2},\mu_{2},\nu_{2}$	The parameters of the controller
$u_{eq}$	The equivalent control input
$u_{sw}$	The switching control input
X	The input of RBFNN
${\hat{u}}_{eq}$	The approximate equivalent control output
$\hat{W}$	The weight of the RBFNN
h(X)	The nonlinear mapping of the RBFNN
$u_{eq}^{*}$	The ideal equivalent control
W	The ideal weight of the RBFNN
ε	The approximation error of the neural network
${\tilde{u}}_{eq}$	The equivalent control output error generated by the neural network estimation
$\tilde{W}$	The weight error of the neural network
$V_{1},V_{2}$	The Lyapunov function
ξ	The parameter of the neural network adaptation law
$u_{x},u_{y}$	The virtual input force of the horizontal subsystem
